# Anti-invasion Effects of Cannabinoids Agonist and Antagonist on Human Breast Cancer Stem Cells

**Published:** 2017

**Authors:** Fatemeh Mohammadpour, Seyed Nasser Ostad, Shima Aliebrahimi, Zahra Daman

**Affiliations:** a *Department of Toxicology and Pharmacology, Faculty of Pharmacy and Poisoning Research Center, Tehran University of Medical Sciences, Tehran, Iran. *; b *Department of Cellular and Molecular Biology, School of Biology, College of Science, University of Tehran, Tehran, Iran.*

**Keywords:** AM251, Breast cancer stem cell, ACEA, MDA-MB-231, Invasion, Cannabinoids

## Abstract

Studies show that cancer cell invasion or metastasis is the primary cause of death in malignancies including breast cancer. The existence of cancer stem cells (CSCs) in breast cancer may account for tumor initiation, progression, and metastasis. Recent studies have reported different effects of cannabinoids on cancer cells via CB1 and CB2 cannabinoid receptors. In the present study, the effects of ACEA (a selective CB1 receptor agonist) and AM251 (a selective CB1 antagonist) on CSCs and their parental cells were investigated. Breast CSCs derived from MDA-MB-231 cell line were sorted and characterized with CD44^+^/CD24^-/low^/ESA^+^ phenotype. It was observed that ACEA decreased CD44^+^/CD24^-/low^/ESA^+^ cancer stem cell invasiveness. Conversely, AM251 increased the invasion by more than 20% (at the highest concentrations) in both MDA-MB-231 and CSCs. Our results did not show any correlation between reduced invasion and cytotoxic effects of the drug. Since one of the main cancer recurrence factors is anti-cancer drugs fail to inhibit CSC population, this observation would be useful for cancer treatment.

## Introduction

The 21-carbon terpenophenolic compounds, cannabinoids, extracted from *Cannabis sativa* (marijuana, hemp plant) have been identified with a wide spectrum of pharmacological effects during the past decades (1). They mimic the effects of endogenous cannabinoids named as endocannabinoids (2). Two cannabinoid receptors are biologically important and have been studied widely: type 1 cannabinoid receptor (CB1) and type 2 cannabinoid receptor (CB2) which belong to a large family of G-protein-coupled receptors (GPCRs) (3, 4). CB1 is predominantly expressed in brain and many peripheral tissues such as ovaries, uterus, testis, adrenal gland, prostate and placenta while CB2 is exclusively expressed in the immune system such as spleen, tonsils, thymus, bone marrow, B-cells, natural killer cells, monocytes, polymorphic mononuclear cells, and neutrophils (5, 6).

Since the first report of the anti-neoplastic activity of cannabinoids (7), it is now known that they can inhibit tumor cell growth by modulating various cell signaling pathways in multiple cancer cells such as lymphoma (8, 9), breast (5, 10), pancreatic, and skin (11, 12). Cannabinoids inhibit cancer cell growth and migration in gastrointestinal tract cancer and induce apoptosis in colorectal cancer cells (13-15). In hepatocellular carcinoma (HCC), the cannabinoids can inhibit growth, proliferation, and invasion of the cancer cells (16, 17). It was also shown that the cannabinoid administration in animals causes the regression of lung adenocarcinomas (7) and thyroid epithelium (18).

Synthetic cannabinoids such as delta-9-tetrahydrocannabinol (THC) (Marinol TM) and its analog nabilone (Cesamet TM) are clinically used in the USA, Canada, and the UK for their palliative effects on chemotherapy-induced nausea and vomiting (19, 20). Rimonabant is also used in the treatment of obesity and related cardiometabolic disease (21).

In most types of solid tumors, morbidity and mortality are attributed to metastasis of invasive tumor cells. Cancer cell metastasis involves sequential processes including actin-myosin cytoskeleton remodeling, extracellular matrix rearrangement by proteolytic enzymes such as serine proteinase, matrix metalloproteinase (MMPs), cathepsin and plasminogen activator, of which MMP-2, MMP-9, and u-plasminogen activator are considered to play crucial roles in basement membranes degradation and to migrate to distant organs through blood and lymphatic circulation. After extravasation, these metastatic cells develop to produce the secondary tumor (22, 23).

Recent studies have revealed the crucial role of cancer stem cells (CSC) in drug resistance and cancer metastasis. CSCs are the subpopulation of tumor cells (0.1-1%) with normal stem cell properties, especially the capability to give rise to a heterogeneous lineage of cancer cells found in tumor mass. Other characteristics such as self-protection, long lifespan, telomerase activation, apoptosis resistance, pluripotency transcription factors expression like Oct4, Nanog, Sox2 as well as survival signaling pathway deregulation (NF-κB, Wnt, Hh, Notch, PI3K, JAK/STAT, BMP, and IGF) have been proposed as CSCs features. It has been reported that CSCs can originate from normal stem cells, progenitor, or differentiated cells that undergo genomic instability mediated through several gene mutations. In addition, it seems that these cells are associated with tumor initiation, progression, metastasis, and recurrence of cancer. However, conventional cancer therapies (like chemotherapy and radiotherapy) target tumor bulk leading to tumor growth inhibition temporarily but show a lack of efficacy to eradicate CSCs. Therefore, CSCs are a promising target for cancer prevention and treatment (24, 25). 

Although the anti-neoplastic activity of cannabinoids has been reported in breast cancer cells, this effect on breast cancer stem cells has not been investigated. In the present study, we evaluated the effect of CB1 agonist and antagonist on proliferation and invasion potential of (CD44^+^/CD24^-/low^/ESA^+^) human breast cancer stem cells which were derived from MDA-MB-231 and their parental cells. Our results indicate that cannabinoids may interfere with invasive cancer stem cells in benefit of cancer eradication.

## Experimental

*Reagents and Antibodies*

**Figure 1:**
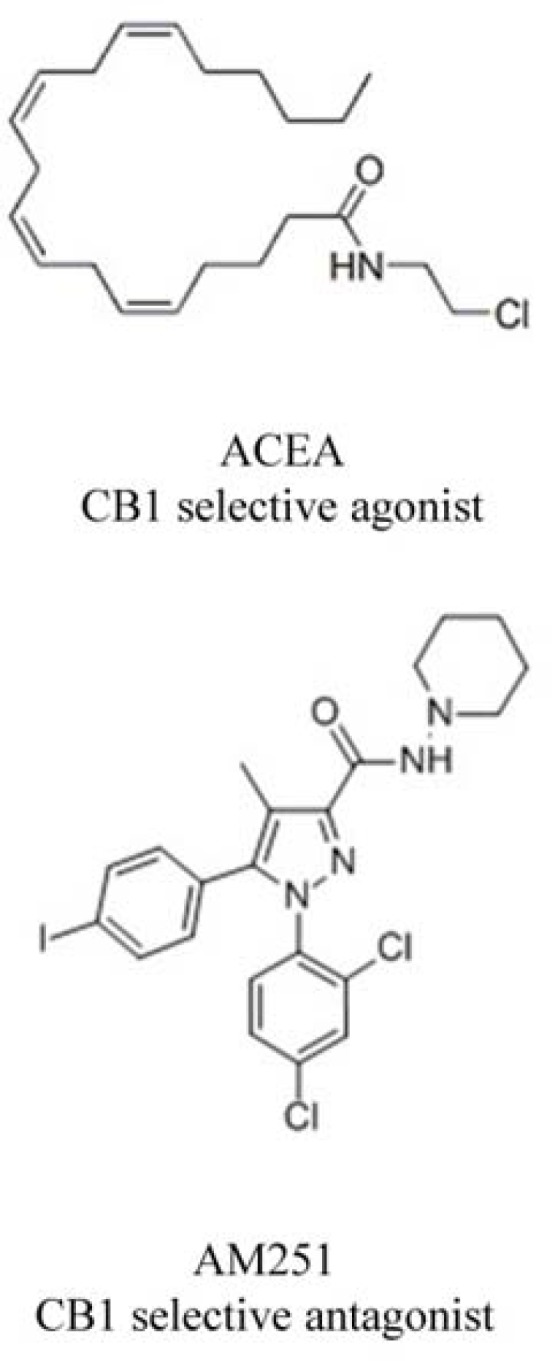
Chemical structure of ligands used in this study

Selective CB1 receptor agonist, arachidonyl-2´-chloroethylamide (ACEA), and selective antagonist of CB1 (N-(Piperidin-1-yl)-5-(4-iodophenyl)-1-(2,4-dichlorophenyl)-4-methyl-1H-pyrazole-3-carboxamide) (AM251) (Figure1) were purchased from Tocris Bioscience (Wiesbaden-Nordenstadt, Germany). AM251 and ACEA were dissolved in dimethyl sulfoxide (DMSO) and ethanol, respectively. Dulbecco’s Modified Eagle’s Medium (DMEM), fetal bovine serum (FBS) and penicillin–streptomycin were purchased from Biosera (East Sussex, UK). Matrigel was obtained from BD Biosciences (Oxford, UK). 3-[4,5-dimethylthiazol-2-yl]-2,5-diphenyltetrazolium bromide (MTT) and DMSO were purchased from Sigma-Aldrich (St. Louis, MO). All other chemicals were of analytical grade and used as received.

**Figure 2:**
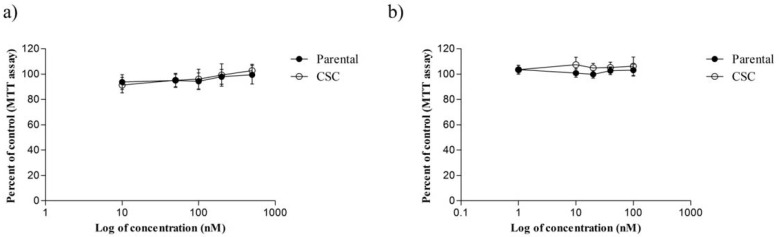
The cytotoxic effect of ACEA and AM251 on MDA-MB-231 cell line and CSCs isolated from it following 48 and 72 h treatment periods, respectively. (a) ACEA (b) AM251. In these assays, 1 × 10^4 ^cells were seeded in 96-well plates followed by incubation overnight. Then, cells were incubated with defined concentrations of ACEA and AM251 for the indicated time periods

**Figure 3.**
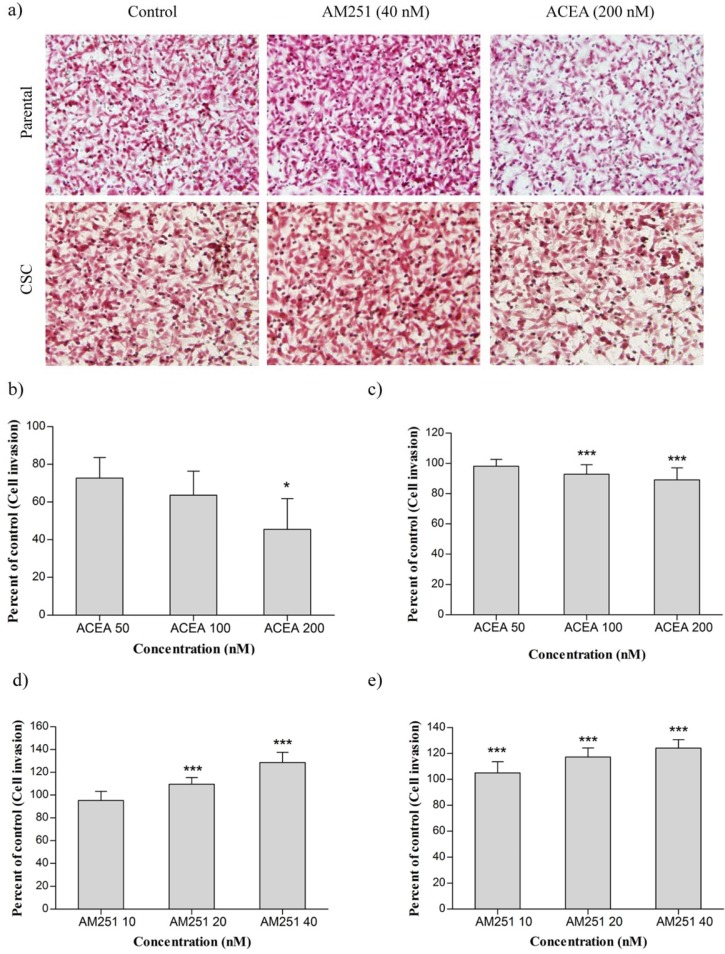
(a) The effect of ACEA and AM251 on invasion of MDA-MB-231 cell line and CSCs isolated from it. (b) MDA-MB-231 cells were treated with ACEA. (c) CSCs were treated with ACEA. (d) MDA-MB-231 cells were treated with AM251. (e) CSCs were treated with AM251. Data were reported as mean ± SD. (* *P *< 0.05; *** *P *< 0.001 relative to vehicle-treated controls

*Cell culture*

MDA-MB-231 (human breast carcinoma cell line) was obtained from Pasteur Institute Cell Bank of Iran (Tehran, Iran). Breast cancer cells were cultured in DMEM supplemented with 10% FBS and penicillin-streptomycin (100 U/mL). The cells were kept under standard cell culture conditions at 37 °C in a humidified atmosphere of 5% CO_2_. The cells were fed fresh media every 3-4 days and subcultured at 80% confluence with 0.05% trypsin–EDTA. 

*CSC Isolation and Characterization*

The human breast cancer stem cells (CD44^+^/CD24^-^/ESA^+^) were sorted and characterized as described previously (26).

*Cell Viability Assay*

The MTT assay was performed to evaluate the effect of ACEA and AM251 on the proliferation of breast cancer cells. 

The cells (1 × 10^4^) were seeded into 96-well plates and incubated for 24 h. Then, they were treated with defined dose of cannabinoid receptor agonist and antagonist for the indicated time periods. Following incubation, the medium was removed and 20 μL of MTT solution (5 mg/mL in PBS) was added for 4 h. To dissolve the purple MTT formazan crystals, 100 μL of DMSO was added to each well and measured spectrophotometrically at 570 nm with a reference wavelength of 690 nm using a multiwell microplate reader (Anthos, UK). Cell viability was calculated as a percentage of untreated control cells.

*Cell Invasion Assay*

Invasion assay was performed using Matrigel-coated polyethylene terephthalate membranes according to the manufacturer’s instructions to evaluate the effect of reagents on the invasive potential of breast cancer cells. The assay was done using transwell inserts (SPL, pore size: 8 μm) preloaded with 32 µL of diluted (1:4) matrigel in serum-free medium and allowed to gel for 6 h at 37 °C. The cells were detached by trypsin and washed three times with culture medium containing 1% FBS. The cell pellet was resuspended in media supplemented with 1% FBS at a density of 5 × 10^4 ^cells/mL with or without the indicated concentration of ACEA and AM251 and seeded on the top of wells. The lower chamber was filled with medium containing 20% FBS as a chemo-attractant. After incubation period for 48-72 h, the adherent cells on the upper surface were gently removed with a cotton swab. Then, the invaded cells on the lower surface of filters were fixed with cold methanol for 30 min, stained with hematoxylin and eosin (Mayer, Germany) and also counted in five randomly selected fields under a light microscope. For each replicate, the results were averaged and compared with negative and positive controls.

*Statistical Analysis*

The results are presented as the mean ± standard deviations for three independent experiments performed at least in triplicate. The reported data were analyzed using SPSS 21.0 software. Comparison of the obtained data for different samples was performed with one-way ANOVA and an appropriate post-test, if necessary. A *P*-value < 0.05 was considered significant.

## Results

*Cytotoxicity Activity of ACEA and AM251 on CSC Derived from MDA-MB-231*

The viability of CSC and MDA-MB-231 parental cells were evaluated in the presence of various concentrations of ACEA (10, 50, 100, 200 and 500 nM) and AM251 (1, 10, 20, 40 and 100 nM) by MTT assay (Figure 2). As shown in Figure 2a, although treatment of MDA-MB-231 and CSCs by ACEA inhibited cell proliferation at lower doses (< 200 nM) as compared with untreated control cells, it was not statistically significant (*P *> 0.05). Further, at 10 nM ACEA decreased CSC viability but it was out of concentration range which was used in invasion assay (*P *< 0.05). Conversely, AM251 treatment increased CSC and their parental cell growth though no significant difference was observed (Figure 2b). On the basis of these results, AM251 and ACEA did not mediate any remarkable inhibition of CSC and MDA-MB-231 proliferation, especially at treated doses used in invasion assay.

*Effect of ACEA and AM251 on Invasion of Human Breast Cancer Cells*

Cannabinoids have shown an inhibitory effect on invasion of some human cancer cells. To examine whether ACEA and AM251 also have the same effect in breast cancer cells, MDA-MB-231 and MDA-MB-231 CSC were treated with ACEA (50, 100 and 200 nM) and AM251 (10, 20 and 40 nM). The treated cells were then evaluated by matrigel invasion assay. 

As shown in Figure 3a, treatment of MDA-MB-231 cells with ACEA could dramatically reduce MDA-MB-231 cell invasion in all concentrations, especially at 200 nM. Our results revealed that treatment of MDA-MB-231 CSCs with ACEA at concentrations of 100 and 200 nM reduced cell invasion that was statistically significant compared with the control group. Nevertheless, ACEA did not affect the cell invasion at 50 nM (Figure 3b). Figures 3c and 3d indicated that invasion of MDA-MB-231 cells increased at 20 and 40 nM of AM251 remarkably. In the case of CSCs, AM251 treatment enhanced invasiveness in a dose-dependent manner relative to untreated cells. 

## Discussion

With advances in cancer biology, the cancer stem cells or tumor-initiating cells were identified as pluripotent cells with a unique capacity of self-renewal being able to differentiate into a heterogeneous population of cells to form tumors. Technical progress over decades led to purification and identification of CSCs based on specific surface markers. Biomarkers such as CD44, CD24, Cytokeratin 5 (CK5), and SOX4 have been proposed for isolation and characterization of breast CSCs (27). It has been shown that breast CSCs are multiple, distinct, and non-overlapping populations co-existing within the tumor mass. Since a minute fraction of tumor cells includes CSCs, their characterization usually mediated through isolation using cell surface markers and development *in-vitro* (28). Most current studies have demonstrated CD44 and CD24 as proposed markers for isolation of CSC subset in breast tumors (29). In our previous study, we isolated CD44^+^/CD24^-/low^ breast CSCs from the main population of MDA-MB-231 cell line using MACS and evaluated the CD44/CD24 expressions by flow cytometry. After isolation, the percentage of subpopulation expressed CD44^+^/CD24^-/low^ biomarkers elevated significantly from 51.10% to 82.24% (26). In addition, it was reported that isolated CSCs exhibit more tumorigenicity *in-vivo* and resistance to conventional chemotherapy and radiotherapy than their parental cells. Therefore, cancer stem cell-based therapies are being investigated as a promising avenue to successful cancer treatment (28).

Recently, investigations clarified that cannabinoids inhibit cell proliferation, induce apoptosis and harness cell migration and angiogenesis in various cancer cells (30). Vara and colleagues found that treatment of hepatocellular carcinoma cells with THC which is the active component of *Cannabis sativa* decreases cell viability and tumor growth via autophagy *in-vitro* and *in-vivo* (31). However, our MTT assay showed that ACEA and AM251 do not have an anti-proliferative effect on CSCs and MDA-MB-231. Thus, no correlation between the anti-invasion effect of cannabinoid agonist ACEA and cytotoxic effects of the drug was found and the specific cannabinoid receptors may be involved (32).

Analyzing the relation between ligand doses in the target zone and biological response obtained are among the most remarkable research fields with many clinical applications. Unlike previous studies on cannabinoids applied AM251 at micromolar doses (33), the present study used nanomolar doses of AM251. Studies indicated that nanomolar doses of cannabinoids may function as growth factor-like molecules in autocrine and/or paracrine manner and accelerate tumor growth (34) while cannabinoid agonists at micromolar concentration reduce tumor cell proliferation in a dose-dependent manner (35).

The results of MTT assay on human MDA-MB-231 cancer cells in the presence of several cannabinoid antagonists illustrated that these compounds increase cell proliferation at nanomolar concentration. Besides, at 10 µM of methanandamide (an agonist of CB receptors and vanilloid receptor 1), cell proliferation significantly increases. These studies showed the proliferative effects of cannabinoids at low doses (32).

Extensive studies have reported that Epithelial-to-Mesenchymal Transition (EMT) promotes invasion and migration of carcinoma cells. Induction of EMT features lead to cell reprogramming and generate cells with CSC properties and invasive potential required for metastatic spread (25). Our data showed that ACEA has an inhibitory effect on CD44^+^/CD24^-^breast cancer stem cell and their parental cell invasion but AM251 increases invasiveness. It is important to mention that CD44, a transmembrane glycoprotein, can interact with hyaluronic acid, fibronectin, fibrinogen, collagen, laminin, fibroblast growth factor-2, osteopontin, and MMPs which is correlated with cell migration (36). Blázquez *et al.* demonstrated that THC administration interferes with MMP2 expression and prevents cell invasion in glioma mouse model (37). In addition, treatment of MDA-MB-231 cells with ACEA inhibits MMP2, VEGF, and cyclooxygenase-2 expression via CB1 receptors which have expressed in MDA-MB-231 cells as confirmed by RT-PCR and western blot (5, 32). Of special note, CB1 cannabinoid receptor is responsible for anti-angiogenic effects and inhibition of cell invasion in human metastatic MDA-MB-231 breast carcinoma cells (32). 

In general, cannabinoid agonists lead to CB1 receptor overexpression however a similar response has been observed in various cancer types (18). Studies demonstrated that increased cannabinoid receptor expression happens upon THC treatment in cancer cells compared to normal cells. The mechanism of this phenomenon has not elucidated yet but it has been recognized that there is an important correlation between cannabinoid receptors and cancer (38). The existence of CB1a and CB1b (splice variants of CB1) could reflect the different response of cannabinoid receptors in malignant and normal cells (39).

In summary, our results clarified that cannabinoid receptor agonist possesses anti-invasion potential in both main population and breast cancer stem cells while AM251 exhibits converse effects. Additionally, ACEA and AM251 did not show any cytotoxicity towards CSCs and MDA-MB-231 parental cells. Considering that most anti-cancer drugs do not eradicate stem cells and only target main population cells, the results disclosed here can be used for prevention of cancer recurrence. 
